# Prevalence and non-invasive predictive models of vitamin D insufficiency in Paraguayan youth: the role of body composition and traditional mate consumption

**DOI:** 10.3389/fnut.2026.1953400

**Published:** 2026-09-11

**Authors:** Anabel Gonzaléz, Camila Miño, Elena Espinoza, Marlene Cano, Milciades Recalde, Carlos Miguel, Dai Ishikawa

**Affiliations:** 1 Department of Biochemistry, Faculty of Health Science, Universidad Santa Clara de Ásis (USCA), Caaguazú, Paraguay; 2 Department of Medicine, Faculty of Health Science, Universidad Santa Clara de Ásis (USCA), Caaguazú, Paraguay; 3 Japan International Cooperation Agency (JICA), Asunción, Paraguay

**Keywords:** body composition, logistic regression, mate consumption, predictive model, traditional habit, vitamin D insufficiency

## Abstract

**Background:**

Vitamin D (VD) insufficiency among youth remains a latent public health concern in Paraguay, and non-invasive, population-specific screening tools are unestablished. This study aimed to identify clinical, body composition, and lifestyle predictors to develop a preliminary, non-invasive risk scoring system for Paraguayan youth.

**Methods:**

Serum 25-hydroxyvitamin D levels, bioelectrical impedance-derived body composition, and lifestyle habits were evaluated in 164 participants (aged 17–35 years) at the Universidad Santa Clara de Asís. Multivariable logistic regression models were constructed after resolving multicollinearity (variance inflation factor <1.40) and underwent internal validation via 1,000 bootstrap resamples.

**Results:**

The overall prevalence of VD insufficiency (<30 ng/mL) was 48.2%. Multivariable analysis revealed an independent contribution of sex-specific muscle mass deviations (Δ Muscle Mass), showing decreased VD levels in both the sarcopenic obesity phenotype and high muscle-low fat athletic phenotype, supporting the skeletal muscle tissue reservoir hypothesis. Among lifestyle factors, full-coverage clothing was an independent risk factor, whereas vigorous physical activity and habitual mate/tereré consumption (≥4 days/week) were significant associated with a lower probability of VD insufficiency. The global predictive scorecard achieved an optimism-corrected area under the receiver operating characteristic curve (optimism-corrected AUC) of 0.714. Crucially, the subgroup model restricted to overweight individuals (BMI ≥ 25 kg/m^2^) demonstrated acceptable exploratory discrimination (optimism-corrected AUC = 0.768, sensitivity 69.2%, specificity 89.7%), serving as a preliminary proof-of-concept assessment for risk stratification.

**Conclusion:**

By integrating body composition dynamics and cultural habits, this cost-effective model provides a preliminary, exploratory non-invasive screening structure for Paraguayan youth that warrants further multi-center prospective validation before clinical implementation.

## 1 Introduction

Vitamin D (VD) insufficiency and deficiency (defined as <30 ng/mL and <20 ng/mL, respectively) are critical global public health concerns (1–4). Beyond classical bone metabolism disorders (1), low serum 25-hydroxyvitamin D [25(OH)D] concentrations have been consistently linked to metabolic dysfunction, including type 1 diabetes (5) and dyslipidemia (6), as well as immune system dysregulation (7) and mental health conditions such as depression (8). Although hypovitaminosis D has historically been associated with obesity and high body mass index (BMI ≥ 25 kg/m^2^) due to volumetric dilution in adipose tissue (4, 6), recent evidence highlights an emerging vulnerability among non-obese, highly active, and athletic young adults (9, 10).

Despite receiving over 2,500 h of annual sunshine (11), Paraguay exhibits a high latent prevalence of VD insufficiency among young individuals (12, 13). Sun exposure behavior in South America is shaped by a complex interplay of environmental factors, skin phototypes, and socio-cultural habits. In Paraguay, these habits uniquely include prolonged outdoor leisure time combined with shaded consumption of mate and tereré, as well as specific clothing choices.

Non-invasive screening tools and predictive risk scores have gained traction as cost-effective alternatives to routine serum 25(OH)D testing in primary healthcare. However, existing scoring systems—such as the ViDDPreS tool developed for young Japanese women (14) or the EVIDENCe-Q questionnaires validated in European adult and pediatric populations (15, 16)—rely heavily on predictor variables calibrated to high-latitude geography, local dietary fortification, and non-Latin American ethnicity. Applying these external screening models to young Paraguayan adults carries a substantial likelihood of misclassification, as they fail to capture local biological, anthropometric, and cultural determinants. Consequently, there remains a critical knowledge gap regarding validated, population-specific non-invasive predictive models tailored to Latin American youth.

To address this limitation, this cross-sectional study aimed to estimate the prevalence of VD insufficiency in a cohort of young Paraguayan adults at the Universidad Santa Clara de Asís (USCA) in Caaguazú, Paraguay, and to identify key clinical, anthropometric, and lifestyle predictors—specifically integrating the culturally deeply rooted habit of mate/tereré consumption. We hypothesized that a non-invasive predictive screening score incorporating anthropometric metrics, skin phototype, and regional lifestyle habits would effectively stratify the risk of VD insufficiency in young Paraguayan adults, offering an exploratory, low-cost preliminary screening structure prior to invasive biochemical assessment.

## 2 Materials and methods

### 2.1 Study population and design

This observational, analytical, cross-sectional study was conducted at the Universidad Santa Clara de Asís (USCA) in Caaguazú, Paraguay. Fieldwork and biological sampling were carried out from March 9 to 13, 2026. Participants were assigned unique numerical codes at enrollment to maintain strict confidentiality. From an initial pool of 166 recruited university students and staff (inclusion criterion: age 17–35 years), two individuals were excluded: one exceeded the age range (57 years old) and one presented incomplete primary demographic records. The final analytical sample comprised 164 participants (mean age: 20.8 ± 3.3 years).

### 2.2 Biochemical analyses and anthropometric assessments

Peripheral venous blood samples were collected from each participant upon enrollment. Serum 25-hydroxyvitamin D [25(OH)D] concentrations were quantified using a dry fluorescence immunoassay kit (25-OH Vitamin D Test Kit, Immunofluorescence; Genrui Biotech Inc., Shenzhen, China) operated on a Quantitative Immunoassay Analyzer (Model FA50; Genrui Biotech Inc.). Total cholesterol and triglycerides were measured via enzymatic colorimetric Trinder methods using commercial assays (Analisa® Ref. 111 and Ref. 459, respectively; Analisa Diagnostica, Brazil). High-density lipoprotein cholesterol (HDL-C) was quantified using a direct assay kit (Ref. EGSHDL-CA; G-cell Biotechnology Co., Ltd., China). All lipid parameters were analyzed on an automated chemistry analyzer (BIOELAB Model AS-120; Nanjing Bioelab Medical Technology Co., Ltd., Nanjing, China; distributed by Janz & Janz, Asunción, Paraguay). Vitamin D insufficiency was defined as serum 25(OH)D <30 ng/mL, and deficiency as <20 ng/mL, in accordance with international clinical guidelines.

Body composition parameters, including skeletal muscle percentage (%) and body fat percentage (%), were measured via bioelectrical impedance analysis (BIA) using a professional body composition monitor (Model: HBF-514C; OMRON Healthcare Co., Kyoto, Japan). Anthropometric measurements, including waist circumference (cm) and neck circumference (cm), were recorded using standard clinical protocols.

To account for sex-specific physiological dimorphism and enable valid comparisons across male and female participants, these anthropometric and body composition variables were standardized using sex-specific median centering. Specifically, continuous variables for muscle mass percentage, body fat percentage, waist circumference, and neck circumference were transformed into delta (Δ) variables by subtracting the respective sex-specific median from each participant’s observed value:

$$\begin{array}{l} \Delta \alpha \;\text{Muscle Mass}\;(\%)=\text{Muscle Mass}\;(\%)\\ -\text{Median}\;{\text{Muscle Mass}}_{\mathrm{sex}}\;(\%) \end{array}$$

$$\Delta \;\text{Body}\;\mathrm{Fat}\;(\%)=\text{Body}\;\mathrm{Fat}\;(\%)-\text{Median Body}\;{\mathrm{Fat}}_{\mathrm{sex}}\;(\%)$$

$$\begin{array}{l} \Delta \;\text{Waist Circumference}\;(\mathrm{cm})=\text{Waist Circumference}\;(\mathrm{cm})\\ -\text{Median}\;{\text{Waist Circumference}}_{\mathrm{sex}}\;(\mathrm{cm}) \end{array}$$

$$\begin{array}{l} \Delta \;\text{Neck Circumference}\;(\mathrm{cm})=\text{Neck Circumference}\;(\mathrm{cm})\\ -\text{Median}\;{\text{Neck Circumference}}_{\mathrm{sex}}\;(\mathrm{cm}) \end{array}$$

This centering approach allows the transformed variables (Δ) to represent relative individual deviations from sex-specific population medians rather than raw unadjusted values, eliminating collinearity with biological sex during multivariable modeling.

### 2.3 Lifestyle questionnaire

Lifestyle habits and sociocultural factors were assessed using a mixed-method approach, combining structured face-to-face interviews with an online questionnaire.

### 2.4 Missing data handling

Prior to statistical modeling, data completeness was verified. Because the single participant with missing questionnaire entries was excluded during initial sample selection, the final analytical dataset (*n* = 164) contained no missing values across any demographic, anthropometric, biochemical, or lifestyle variables. Therefore, a complete-case analysis was performed without the need for statistical imputation algorithms.

### 2.5 Variable binarization and threshold selection

For lifestyle variables with ordinal response options derived from the Lifestyle Questionnaire (e.g., habitual mate/tereré consumption frequency and degree of sun exposure according to usual clothing), binarization thresholds were determined through an exploratory screening protocol during preliminary analysis. Candidate split-points were created by sequentially shifting the threshold along the category gradient (e.g., for mate/tereré intake: Contrast 1: 0 days/week = 1 vs. ≥1 day/week = 0; Contrast 2: ≤1 day/week = 1 vs. ≥2 days/week = 0). Split-points with extreme sample size imbalances were pre-screened out to prevent numerical instability and sparse-data bias in multivariable modeling. Among the remaining candidate contrasts, those demonstrating statistically significant associations (*p* < 0.05) in both the primary Chi-square (*χ*^2^) test and the point-biserial correlation analysis with consistent directionality were selected as candidate variables for multivariable logistic regression modeling.

### 2.6 Statistical analyses

Statistical analyses were performed using Microsoft Excel and the statistical software EZR (Easy R) (17), based on R Commander (version 2.9-5) and running on R (version 4.5.2; The R Foundation for Statistical Computing, Vienna, Austria). Multivariable logistic regression modeling was conducted to identify predictors of vitamin D insufficiency. To evaluate model optimism and prevent overfitting, internal validation was performed using 1,000 bootstrap resamples with the ‘boot’ and ‘pROC’ packages in R. Model discrimination was assessed using the apparent and optimism-corrected Area Under the Receiver Operating Characteristic Curve (AUC). Two-tailed *p* < 0.05 was considered statistically significant. To ensure full transparency and scientific reproducibility, all underlying R scripts, data tables, and statistical command logs have been deposited in a public repository.^1^

Bivariate analyses were first performed utilizing Pearson’s correlation coefficient and Welch’s *t*-test for continuous variables, and Chi-square tests or Fisher’s exact tests for categorical associations with VD insufficiency. To evaluate non-linear joint body composition effects, participants were stratified into four distinct phenotypes based on sex-specific medians of muscle and fat mass (Table 1), with interaction terms evaluated via Type III analysis of variance (ANOVA).

**Table 1:** Association of body composition phenotypes with vitamin D levels.

Body fat	Muscle mass	*n*	Vitamin D (ng/mL)	VD Insufficient	VD sufficient
			Mean ± SD^†^	*n* (%)^*^	*n* (%)
High	High	17	34.2 ± 10.7^a^	7 (41.2%)	10 (58.8%)
	Low	66	29.8 ± 8.0	32 (48.5%)	34 (51.5%)
Low	High	66	30.2 ± 9.0^b^	33 (50.0%)	33 (50.0%)
	Low	15	32.7 ± 9.9	7 (46.7%)	8 (53.3%)

^†^*p*-value for the interaction term of the two-way ANOVA (Type III) = 0.057 (indicates a trend toward a significant interaction between body fat and muscle mass on continuous vitamin D levels).

^*^p-value obtained using the Chi-square test for the frequency distribution of vitamin D insufficiency (*n* = 79: 7, 32, 33, and 7) among the four body composition groups (*χ*^2^ = 32.9, Cramer’s *V* = 0.65, *p* < 0.001).

^a^*p* = 0.140 when comparing muscle mass (high vs. low) within the high body fat group (Welch’s *t*-test).

^b^*p* = 0.407 when comparing muscle mass (high vs. low) within the low body fat group (Welch’s *t*-test).

Candidate predictor selection for multivariable logistic regression was guided by combining initial bivariate significance (*p* < 0.05), biological relevance to VD metabolism, Akaike Information Criterion (AIC) minimization, and variance inflation factor (VIF < 1.35) diagnostics to preserve model parsimony and avoid collinearity (18). Specifically, BMI was retained as a baseline adjusting covariate to control for general adiposity (4, 8, 19). Crucially, sex-specific median centered Δ Muscle Mass was designated as a primary predictor of interest to directly test the skeletal muscle tissue reservoir hypothesis, representing the joint dynamics of muscle and fat mass. The multivariable model yielded regression coefficients (*b*) and odds ratios (OR) with 95% confidence intervals (95% CI).

### 2.7 Scorecard construction and internal validation

The individual predicted probability (*P*) of vitamin D insufficiency is calculated using the standard logistic function:

$$P(\mathrm{VD}\;\text{insufficiency})=\frac{1}{1+e^{-\text{logit}(P)}}$$

Where the linear predictor (logit(*P*)) for the Total Subjects Model (*n* = 164, Intercept = −1.638) is expressed as:

$$\begin{array}{l} \text{logit}(P)=-1.638+0.982\;(\text{Female})\\ a+0.012\;(\mathrm{BMI})+0.063(\Delta \alpha \;\text{Muscle Mass}) \end{array}$$

+1.276(Full Clothing)−0.898(VigorousPA)

+1.027(infrequent Mate)

And for the Overweight Subgroup Model (*n* = 68, Intercept = −4.298):

$$\begin{array}{l} \text{logit}(P)=-4.298+0.137\;(\mathrm{BMI})+0.164\;(\Delta \;\text{Muscle Mass})\\ +1.517\;(\text{Full Clothing}) \end{array}$$

−1.331(VigorousPA)+1.228(Infrequent Mate)

Here, continuous variables (BMI in kg/m^2^ and Δ Muscle Mass in %) are multiplied directly by their respective values, and binary parameters are coded as 1 if present and 0 if absent: Sex (Female = 1, Male = 0), Degree of sun exposure according to usual clothing (represented as Full Clothing in equations: Full body coverage = 1, Little/no coverage = 0), Vigorous physical activity (represented as Vigorous PA in equations: Active ≥10 min/day = 1, Inactive = 0), and Frequency of mate/tereré consumption (represented as Infrequent Mate in equations: Consumption ≤3 days/week = 1, ≥4 days/week = 0).

To construct a user-friendly clinical scorecard, the multivariable *β*-coefficients from these logistic regression models were mathematically transformed into simplified integer weights by dividing each coefficient by the baseline reference coefficient of Δ Muscle Mass (*β* = 0.063 for the total population, and *β* = 0.164 for the overweight model) and rounding to the nearest integer (20). Individual total risk scores were calculated for each participant by summing the assigned integer points corresponding to their present risk factors.

To evaluate internal goodness-of-fit prior to internal validation, apparent model calibration (calibration slope and intercept) was assessed directly within the development dataset by regressing observed outcomes on model linear predictors (logits). Additionally, to evaluate model stability and address potential performance overestimation (optimism) arising from the development dataset, internal validation of the original multivariable logistic regression models was performed using 1,000 bootstrap resamples with the ‘boot’ and ‘pROC’ packages in R. Optimism-corrected Area Under the Receiver Operating Characteristic Curves (AUC) were thereby derived (21, 22).

The discrimination capacity of the resulting integer-based total risk scores was subsequently assessed using univariate ROC analysis, alongside the calculation of optimal cutoff thresholds, sensitivity, specificity, positive predictive value (PPV), and negative predictive value (NPV). Finally, participants were stratified into distinct risk categories based on their total scores, and a Chi-square test for trend was applied to validate risk progression.

## 3 Results

### 3.1 Prevalence of VD insufficiency

The overall mean serum VD level among participants (*n* = 164) was 30.7 ± 9.0 ng/mL (Supplementary Table S1). Notably, 48.2% (*n* = 79) of the study population presented with VD insufficiency (<30 ng/mL), underscoring a substantial prevalence within this cohort. Mean serum VD levels were significantly lower in the VD-insufficient group compared to the VD-sufficient group (*n* = 85; 23.4 ± 4.6 ng/mL vs. 37.5 ± 6.4 ng/mL, Welch’s *t*-test *p* < 0.001).

### 3.2 Primary factors significantly associated with VD insufficiency

To identify potential predictors for the VD insufficiency risk score model, bivariate analyses were performed using Pearson’s correlation coefficient (*r*) and the Chi-square test (*χ*^2^; Supplementary Table S1).

#### 3.2.1 Sex

Females exhibited significantly lower VD levels than males (29.9 ± 9.1 vs. 34.1 ± 7.8 ng/mL; *p* < 0.01, Welch’s *t*-test) and a higher prevalence of VD insufficiency (53.1% vs. 26.5%; *χ*^2^ = 8.1, *p* < 0.005). Point-biserial correlation (Male = 0, Female = 1) confirmed this negative association (*r* = −0.191, *p* < 0.05).

#### 3.2.2 BMI

While overall BMI showed no significant linear correlation with VD levels (*r* = −0.070, *p* = 0.356), a significant negative correlation emerged exclusively among overweight subjects (*n* = 68; *r* = −0.268, *p* < 0.05). No such association was observed in the normal-weight subgroup (*r* = 0.035, *p* = 0.735). Categorically, neither mean VD levels nor insufficiency prevalence differed significantly between the two subgroups.

### 3.3 Primary anthropometric factors and sex-based confounding

Bivariate analyses initially showed significant correlations between crude anthropometrics and serum VD: Muscle Mass correlated positively overall, while Body Fat correlated inversely within the overweight subgroup (Supplementary Table S1). However, biological sex severely confounded these crude metrics, exhibiting strong collinearity with Body Fat (*r* = 0.654), Muscle Mass (*r* = −0.832), Waist Circumference (*r* = −0.423), and Neck Circumference (*r* = −0.620; *p* < 0.001 for all; coded as Male = 0, Female = 1). To neutralize this confounding, these parameters were transformed into sex-specific median-centered delta (Δ) deviations, calculated by subtracting the respective sex-specific medians derived from the study population (see Supplementary Table S1 for detailed formulas and reference values). This transformation successfully eliminated sex-bias for Δ Body Fat, Δ Muscle Mass, and Δ Neck Circumference. In the overweight cohort, only Δ Body Fat maintained a significant inverse correlation with VD levels (*r* = −0.268, *p* < 0.05), whereas Δ Muscle Mass displayed no significant bivariate linear associations, and Δ Neck Circumference exhibited no statistically significant associations in any sub-analysis (Supplementary Table S1).

### 3.4 Collinearity and behavior of delta (Δ) variables

Overall BMI was highly collinear with crude body metrics and the newly transformed difference variables including Δ Body Fat (*r* = 0.864, *p* < 0.001), Δ Muscle Mass (*r* = −0.326, *p* < 0.005), and Δ Waist Circumference (*r* = 0.728, *p* < 0.001; Supplementary Table S1). Crucially, however, unlike their crude counterparts, the difference variables Δ Body Fat and Δ Muscle Mass showed no significant associations with biological sex. This mathematically confirms that transforming these tissue parameters into sex-specific median deviations successfully and completely eliminated baseline gender bias within these components.

### 3.5 Selection of Δ Muscle Mass and BMI as baseline predictors

To establish a clinically applicable and parsimonious risk scorecard, we systematically evaluated the candidate models presented in Supplementary Table S2. While Model F (Sex, Δ Body Fat, and Δ Muscle Mass) yielded a marginally superior statistical fit (AIC = 225.3, omnibus *p* = 0.020), Model D (AIC = 225.7, omnibus *p* = 0.024) was selected as the final baseline structure. This decision was guided by the need to balance statistical parsimony with the representation of our core biological hypotheses.

#### 3.5.1 Δ Muscle Mass (%)

Δ Muscle Mass was retained not as a mere adjusting covariate, but as a primary explanatory predictor representing the central hypothesis of this study—the skeletal muscle tissue reservoir dynamics. Although its linear main effect did not reach statistical significance in the overall model (Model D: OR = 1.03, 95% CI: 0.97–1.09, *p* = 0.298), it demonstrated strong statistical independence (VIF = 1.15). Crucially, as detailed in Table 1, our body composition phenotype analysis revealed that the biological impact of muscle mass is non-linear and conditional on concomitant body fat. Retaining Δ Muscle Mass in the baseline model was therefore indispensable for capturing these complex musculoskeletal dynamics, which were ultimately validated by its highly significant independent contribution within the overweight-specific model (OR = 1.18, *p* < 0.05; Table 2).

**Table 2:** Multivariate logistic regression models and score assignment for the prediction of vitamin D insufficiency in the total and overweight populations.

Variables	Total subjects (*n* = 164)				Overweight subjects (*n* = 68)			
	OR (95% CI)	Estimate	VIF	*p*-value	OR (95% CI)	Estimate	VIF	*p*-value
		*β*-value				*β*-value		
Intercept (*β*_0_)	0.19 (0.02–2.17)	−1.638	–	0.183	0.01 (0.00–1.97)	−4.298	–	0.091
Sex (male vs. female)	2.67 (1.06–6.72)	0.982	1.05	<0.05	–	–	–	–
BMI (kg/m^2^)	1.01 (0.93–1.11)	0.012	1.14	0.794	1.15 (0.97–1.36)	0.137	1.06	0.120
Δ Muscle Mass^†^	1.07 (1.00–1.14)	0.063	1.23	0.052	1.18 (1.04–1.34)	0.164	1.31	<0.05
Degree of sun exposure according to usual clothing	3.58 (1.59–8.08)	1.276	1.10	<0.005	4.56 (1.32–15.8)	1.517	1.11	<0.05
Vigorous physical activity (≥10 consecutive min in the last 7 days)	0.41 (0.20–0.82)	−0.898	1.07	<0.05	0.26 (0.08–0.89)	−1.331	1.10	<0.05
Frequent consumption of mate/tereré (>500 mL/day, ≤3 days/week)	2.79 (1.31–5.93)	1.027	1.10	<0.01	3.41 (0.98–11.9)	1.228	1.11	0.054
Omnibus test (*p*-value)^*^	<0.001				<0.001			
Optimism-corrected AUC^$^	0.714				0.768			

OR *=* Odds Ratio; 95% CI = 95% Confidence Interval; VIF = Variance Inflation Factor; AUC = Area Under the ROC Curve.

^†^Δ Muscle Mass values represent individual deviations from sex-specific population medians.

^*^The Omnibus test evaluated the model coefficients using the *χ*^2^ likelihood ratio test.

^$^Internal validation was conducted using 1,000 bootstrap resamples. For the Total Subjects model, the apparent AUC was 0.748 with an optimism of 0.034. For the Overweight Subjects model, the apparent AUC was 0.816 with an optimism of 0.048. Applying total-population sex-specific medians to the overweight subgroup (*n* = 68) preserved sample size consistency and guaranteed a statistically superior model (AUC = 0.816, AIC = 82.9, *p* < 0.001 vs. group-specific centering: AUC = 0.760, AIC = 91.7, *p* = 0.02).

#### 3.5.2 BMI

In contrast, BMI was deliberately retained as a baseline adjusting covariate to control for general adiposity and volume dilution effects (4, 6, 19). While BMI was not statistically significant in the general model (Model D: OR = 1.01, 95% CI: 0.94–1.10, *p* = 0.737), including it bypassed the severe multicollinearity associated with crude tissue-level parameters (VIF = 1.11). Furthermore, this structural integration ensured direct analytical comparability with the stratified overweight-specific cohort, where the metabolic and anthropometric pathways of both BMI and Δ Muscle Mass became highly pronounced.

### 3.6 Association of body composition phenotypes with VD levels

To evaluate the interaction between body fat and muscle mass, participants were categorized into four phenotypes using sex-specific medians (Table 1).

Two-way ANOVA revealed a trend toward a significant interaction on continuous VD levels (*p* = 0.057), confirming that the biological impact of muscle mass is non-linear and conditional on concomitant body fat. Within the high-body-fat stratum, subjects with “high” muscle mass presented higher mean VD levels compared to those with “low” muscle mass (34.2 ± 10.7 ng/mL vs. 29.8 ± 8.0 ng/mL, *p* = 0.140). Conversely, this relationship was reversed within the “low” body fat stratum, subjects with “low” muscle mass exhibited higher VD levels than those with “high” muscle mass (32.7 ± 9.9 ng/mL vs. 30.2 ± 9.0 ng/mL, *p* = 0.470). Although these specific pairwise subgroup comparisons did not reach strict statistical significance, this mixed directional pattern reinforces the presence of a complex interaction.

Chi-square analysis demonstrated a highly significant association between these four phenotypes and the prevalence of VD insufficiency (*χ*^2^ = 32.9, *p* < 0.001), confirming that the simultaneous joint configuration of body composition phenotypes exerts a determining influence on VD status.

### 3.7 Selection of lifestyle factors and traditional habits

#### 3.7.1 Sun exposure via habitual clothing

Subjects with “Full coverage/Covers most of the body” presented significantly lower VD levels than those with “Covers very little/Little to no coverage” (27.1 ± 8.2 vs. 32.1 ± 9.0 ng/mL; *p* < 0.005). Point-biserial correlation (“Covers very little/Little to no coverage” = 0, “Full coverage/Covers most of the body” = 1) confirmed this negative association (*r* = −0.244, *p* < 0.01). Furthermore, greater body coverage was strongly associated with a higher prevalence of VD insufficiency (68.9% vs. 40.3%; *χ*^2^ = 10.7, *p* < 0.005; Table 3).

**Table 3:** Association between lifestyle factors and vitamin D levels.

Variables	Category or unit (0/1)^*^	VD value (ng/mL)	Welch’s *t*-test	Pearson correlation coefficient		VD insufficiency (<30 ng/mL)	VD sufficiency (≥30 ng/mL)	Chi-square test		
		Mean ± SD	*p*-value	Pearson’s *r*	*p*-value	*n* (%)	*n* (%)	*χ* ^2^	Cramer’s *V*	*p*-value
Average daily time of outdoor unprotected sun exposure	≥60 min/day (0)	33.4 ± 9.0	0.199	−0.106	0.178	6 (31.6%)	13 (68.4%)	2.4	0.120	0.124
	<60 min/day (1)	30.4 ± 9.0				73 (50.3%)	72 (49.7%)			
Average weekly indoor academic activity hours	≥35 h/week (1)	31.3 ± 9.5	0.311	−0.078	0.320	44 (46.3%)	51 (53.7%)	0.3	0.044	0.577
	<35 h/week (0)	29.9 ± 9.0				35 (50.7%)	34 (49.3%)			
Time period of peak unprotected sun exposure	Before 10:00/after 15:00 (1)	31.3 ± 9.0	0.461	0.058	0.461	35 (48.6%)	37 (51.4%)	0.01	0.001	0.920
	10:00–15:00 (0)	30.3 ± 9.1				44 (47.8%)	48 (52.2%)			
Use of sunscreen with SPF ≥ 30 on exposed areas	Yes (1)	31.8 ± 8.4	0.200	−0.099	0.208	31 (44.3%)	39 (55.7%)	0.74	0.067	0.390
	No (0)	30.0 ± 9.4				48 (51.1%)	46 (48.9%)			
Degree of sun exposure according to usual clothing^#^	Covers very little/little to no coverage (0)	32.1 ± 9.0	<0.005	−0.244	<0.01	48 (40.3%)	71 (59.7%)	10.7	0.255	<0.005
	Full coverage/covers most of the body (1)	27.1 ± 8.2				31 (68.9%)	14 (31.1%)			
Consumption of fish	≥1 time/week (0)	31.6 ± 8.7	0.359	−0.071	0.364	22 (38.6%)	35 (61.4%)	3.2	0.140	0.073
	Never (1)	30.3 ± 9.1				57 (53.3%)	50 (46.7%)			
Consumption of milk, yogurt, and cheese	≥2 times/week (0)	30.9 ± 8.7	0.684	−0.032	0.680	58 (48.3%)	62 (51.7%)	0.01	0.005	0.945
	≤1 time/week (1)	30.2 ± 9.1				21 (47.7%)	23 (52.3%)			
Vitamin D supplement intake	Yes (0)	29.3 ± 6.6	0.309	0.064	0.414	11 (50.0%)	11 (50.0%)	0.03	0.014	0.854
	No (1)	31.0 ± 9.3				68 (47.9%)	74 (52.1%)			
Light/Moderate physical activity (≥10 consecutive min in the last 7 days)^†^	Yes (1)	31.8 ± 9.4	<0.05	0.166	<0.05	49 (45.0%)	60 (55.0%)	1.4	0.091	0.246
	No (0)	28.6 ± 8.1				30 (54.5%)	25 (45.5%)			
Vigorous physical activity (≥10 consecutive min in the last 7 days)^†#^	Yes (1)	33.1 ± 8.6	<0.005	0.245	<0.005	28 (36.4%)	49 (63.6%)	8.1	0.222	<0.005
	No (0)	28.7 ± 8.9				51 (58.6%)	36 (41.4%)			
Daily sedentary time (hours/day)	<6 h (0)	30.3 ± 9.1	0.638	0.060	0.444	38 (53.5%)	33 (46.5%)	1.4	0.094	0.231
	≥6 h (1)	31.0 ± 9.0				41 (44.1%)	52 (55.9%)			
Tobacco smoking habit	Yes (1)	30.6 ± 9.0	0.539	0.050	0.522	75 (49.7%)	76 (50.3%)	1.7	0.102	0.191
	No (0)	32.3 ± 8.9				4 (30.8%)	9 (69.2%)			
Frequency of alcohol consumption	≤1 time/month (0)	30.4 ± 9.3	0.406	0.062	0.431	65 (52.8%)	58 (47.2%)	4.3	0.162	<0.05
	≥2 times/month (1)	31.7 ± 8.2				14 (34.1%)	27 (65.9%)			
Frequency of consumption of mate/tereré (>500 mL/day)^#^	≥2 days/week (0)	31.8 ± 9.2	<0.05	−0.157	<0.05	47 (44.3%)	59 (55.7%)	1.8	0.104	0.184
	≤1 day/week (1)	28.8 ± 8.5				32 (55.2%)	26 (44.8%)			
	≥4 days/week (0)	32.7 ± 10.1	0.057	−0.159	<0.05	21 (36.8%)	36 (63.2%)	4.5	0.165	<0.05
	≤3 days/week (1)	29.7 ± 8.3				58 (54.2%)	49 (45.8%)			

^*^Lifestyle variables were dichotomized and dummy-coded as (0) or (1) for correlation analysis.

^†^Vigorous physical activity was defined as engaging in strenuous exercise (e.g., running, soccer, strength training) for more than 10 consecutive minutes during the last 7 days. Continuous walking or slow cycling was categorized as light/moderate physical activity.

^#^Based on the results of Chi-square test, three factors (Degree of sun exposure according to usual clothing), Vigorous physical activity (>10 consecutive min in the last 7 days), and Frequency of consumption of mate o tereré (>500 mL/day, ≥4 días/week) were included in the multivariable logistic regression model.

#### 3.7.2 Physical activity

Daily engagement in physical activity for at least 10 consecutive minutes during the 7 days preceding the survey was evaluated. For light/moderate physical activity, active subjects presented significantly higher continuous serum VD levels compared to inactive ones (31.8 ± 9.4 vs. 28.6 ± 8.1 ng/mL; *p* < 0.05) and point-biserial correlation (No = 0, Yes = 1; *r* = 0.166, *p* < 0.05). However, no statistically significant categorical association was observed with the prevalence of VD insufficiency (*χ*^2^ = 1.4, *p* = 0.246).

Conversely, vigorous physical activity demonstrated robust positive associations with VD levels in both continuous (33.1 ± 8.6 vs. 28.7 ± 8.9 ng/mL; *p* < 0.005) and point-biserial analyses (No = 0, Yes = 1; *r* = 0.245, *p* < 0.005). Vigorous activity was also strongly associated with a significantly lower prevalence of VD insufficiency (36.4% vs. 58.6%; *χ*^2^ = 8.1, *p* < 0.005). Consequently, due to its superior categorical association and statistical power, vigorous physical activity was selected as the baseline lifestyle predictor for the subsequent multivariate logistic regression model.

#### 3.7.3 Mate or tereré consumption

The impact of this traditional habit was evaluated based on a minimum daily intake of >500 mL. Initial stratification at a threshold of ≥2 days/week showed significantly higher continuous serum VD levels compared to infrequent consumption of ≤1 day/week (31.8 ± 9.2 vs. 28.8 ± 8.5 ng/mL; *p* < 0.05). Point-biserial correlation (≥2 days/week = 0, ≤1 day/week = 1) confirmed this inverse association (*r* = −0.157, *p* < 0.05). However, this lower threshold showed no significant categorical association with the prevalence of VD insufficiency (44.3% vs. 55.2%; *χ*^2^ = 1.8, *p* = 0.184).

In contrast, the higher consumption threshold of ≥4 days/week demonstrated more robust and consistent associations. Subjects consuming mate/tereré ≥4 days/week presented higher mean VD levels compared to those consuming ≤3 days/week (32.7 ± 10.1 vs. 29.7 ± 8.3 ng/mL; *p* = 0.057). Point-biserial correlation (≥4 days/week = 0, ≤3 days/week = 1) confirmed a significant inverse association (*r* = −0.159, *p* < 0.05). Furthermore, categorical analysis revealed that only 36.8% of subjects in the ≥4 days/week group presented VD insufficiency compared to 54.2% in the ≤3 days/week group (*χ*^2^ = 4.5, *p* < 0.05). Consequently, the ≥4 days/week threshold was selected for inclusion in the subsequent multivariate logistic regression model.

### 3.8 Multivariate logistic regression models and predictive score construction

To construct the predictive risk scorecard, multivariate logistic regression models were developed by integrating the three baseline predictors (Sex, BMI, and sex-specific median-centered Δ Muscle Mass), alongside the three selected lifestyle variables (sun exposure via habitual clothing, vigorous physical activity, and frequent mate/tereré consumption (≥4 days/week)). Separate configurations were evaluated for the total population and the high-risk subgroup of overweight subjects (Table 2).

Both final models demonstrated acceptable mathematical stability, with all variance inflation factors (VIF) remaining well below 1.40, effectively ruling out multicollinearity bias. The omnibus tests for both configurations were highly significant (*p* < 0.001), confirming the validity of these combined predictors.

In the total population, Sex (OR = 2.67, *p* < 0.05), full body clothing coverage (OR = 3.58, *p* < 0.005), and infrequent mate/tereré consumption (OR = 2.79, *p* < 0.01) were independent risk factors for VD insufficiency, while Δ Muscle Mass showed a strong positive trend toward risk (OR = 1.07, *p* = 0.052). Conversely, vigorous physical activity was associated with a significantly reduced odds of VD insufficiency (OR = 0.41, *p* < 0.05).

Conversely, in the initial model for overweight subjects, “Sex” completely lost its statistical significance (*p* = 0.344, data not shown). To optimize parsimony and avoid overfit under the events-per-variable (EPV) guidelines (23–25) for this modest sample size (29 events), Sex was definitively excluded. This simplification reduced the Akaike Information Criterion (AIC) from 83.9 in the 6-factor model to a more favorable 82.9 in the final 5-factor model.

Within this finalized overweight model, the metabolic and behavioral risk factors significantly intensified: Δ Muscle Mass doubled its strength of association (OR = 1.18, *p* < 0.05), full body clothing coverage increased its risk profile (OR = 4.56, *p* < 0.05), and a strong trend toward an increased risk was observed with infrequent mate/tereré consumption, although statistical significance was not formally reached (OR = 3.41, *p* = 0.054). Correspondingly, vigorous physical activity was inversely associated with VD insufficiency, corresponding to a 74% lower odds of VD insufficiency (OR = 0.26, *p* < 0.05).

Ultimately, the general model achieved an acceptable discrimination capacity with an optimism-corrected AUC of 0.714 (apparent AUC = 0.748). Crucially, when restricted to the overweight subgroup, the discriminative capacity was higher, yielding an optimism-corrected AUC of 0.768 (apparent AUC = 0.816). These findings indicate that while the scorecard achieves acceptable exploratory discrimination in this subgroup, it should be interpreted strictly as an exploratory, proof-of-concept pilot assessment awaiting prospective validation in independent cohorts rather than a definitive diagnostic instrument. An assessment of apparent model calibration evaluated directly within the development dataset yielded an apparent calibration slope of 1.000 and an apparent calibration intercept of 0.000 in both the primary cohort and the overweight subgroup, confirming optimal internal alignment of raw predicted probabilities prior to optimism adjustment.

### 3.9 Creation of the preliminary scoring system and discriminative capacity validation

Based on the *β*-coefficients derived from the final multivariate models (Table 2), a simplified integer scoring system (scorecard) was constructed to estimate the clinical risk of VD insufficiency (Table 4). For both models, integers were assigned using the *β*-coefficient of Δ Muscle Mass as the baseline reference (*β* = 0.063 → 1 point for the total population, and *β* = 0.164 → 1 point for the overweight model).

**Table 4:** Scorecard for predicting the risk of vitamin D insufficiency.

Variables	Total subjects			Overweight subjects (BMI ≥ 25)		
	Response	Assigned value^*^	Score	Response	Assigned value^*^	Score
Sex	Female	16		–	–	–
	Male	0		–	–	–
BMI (kg/m^2^)		×0.2			×0.8	
Muscle mass (%)	Female	−25.5		Female	−25.5	
	Male	−38.7		Male	−38.7	
Sun exposure and clothing	Covers most of the body	20		Covers most of the body	9	
	Covers very little/little to no coverage	0		Covers very little/little to no coverage	0	
Vigorous physical activity (≥10 consecutive min in the last 7 days)	Yes	−14		Yes	−8	
	No	0		No	0	
Frequency of consumption of mate/tereré (>500 mL/day, ≥4 days/week)	Yes	0		Yes	0	
	No	16		No	7	
		Total score			Total score	

^*^The “Assigned Value” variable represents the integer score of *β*-value assigned to each factor in Table 2, calculated by simplifying the *β* coefficients. Points were assigned using the *β* of ‘Muscle Mass Difference’ as the baseline reference. Calculation: Sum the points of each variable. For BMI, multiply the value (kg/m^2^) by the indicated factor (×0.2 or ×0.8).

Univariate logistic regression analysis demonstrated that both developed scorecards were potent and highly significant predictors of VD insufficiency (Omnibus test *p* < 0.001 for both configurations; Table 5). In the total population, each one-point increase in the score substantially multiplied the risk of presenting with VD insufficiency (OR *=* 1.07; 95% CI: 1.04–1.10). Similarly, in the overweight subgroup, the simplified score maintained a robust and independent association with a deficient VD status (OR *=* 1.21; 95% CI: 1.10–1.34).

**Table 5:** Diagnostic capacity and predictive validation of the total score using univariate logistic regression for vitamin D insufficiency.

Population	OR (95% CI)	*p*-value^*^	Omnibus test^†^	Apparent AUC	Sensitivity	Specificity
Total (*n* = 164)	1.07 (1.04–1.10)	<0.001	<0.001	0.746	56.4%	83.5%
Overweight (*n* = 68)	1.21 (1.10–1.34)	<0.001	<0.001	0.809	69.2%	89.7%

OR *=* Odds Ratio; 95% CI = 95% Confidence Interval; AUC = Area under the ROC curve.

^*^The coefficient and *p*-value correspond to the total score, which was calculated by simplifying and converting the *β* coefficients presented in Table 2 into integers, following the model and instrument detailed in Table 4.

^†^*p*-value from the omnibus test based on the Chi-square statistic for the global evaluation of the model.

Regarding diagnostic accuracy parameters, the scorecard applied to total subjects exhibited notable discrimination (AUC = 0.746), with a sensitivity of 56.4% and a specificity of 83.5%. Crucially, despite the minor mathematical translation loss inherent to integer simplification, the clinical performance of the instrument demonstrated favorable discrimination when restricted to overweight subjects, yielding an AUC = 0.809. This analytical improvement was reflected in an increased sensitivity (69.2%) while maintaining a specificity of 89.7%. These findings indicate that this non-invasive screening scorecard demonstrates higher discriminative performance when evaluated within the overweight subgroup, serving as a preliminary framework for targeted risk stratification.

### 3.10 Risk categorization and clinical validation via serum VD levels

**Figure 1:**
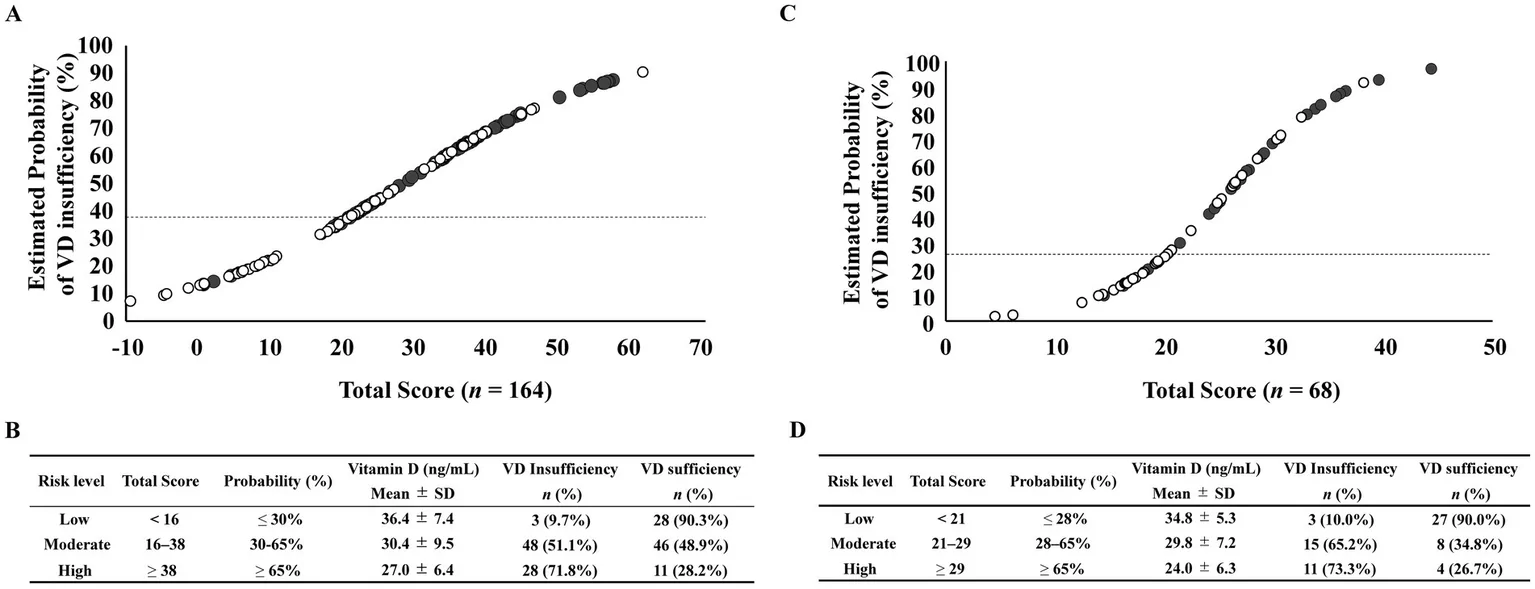
Relationship between the total score and the estimated probability of risk for vitamin D insufficiency and association between risk levels and serum vitamin D status **(A,B)**. Results for the overall population (*n* = 164). **(C,D)** Results for the overweight population (*n* = 68). In panels **(A,C)**, circles represent individual subjects (closed circles ●: VD insufficiency <30 ng/mL; open circles ○: VD sufficiency ≥30 ng/mL). The dashed line indicates the optimal cutoff threshold determined by the logistic regression model [39.0% in panel **(A)** and 28.6% in panel **(C)**]. Panels **(B,D)** show the distribution of VD insufficiency according to the three established risk levels. Strong linear associations were observed between risk levels and VD status in both the overall (*χ*^2^ = 27.43, *p* < 0.001; *p*-for-trend <0.001) and overweight populations (*χ*^2^ = 26.64, *p* < 0.001; *p*-for-trend <0.001).

To facilitate clinical application, the population was stratified into three risk levels (Low, Moderate, and High) based on the estimated probabilities from the logistic models (Figure 1).

In Total Subjects (Figures 1A,B), the scoring system demonstrated clear risk stratification. Within the Low Risk category, 90.3% of subjects presented sufficient VD status, with a mean serum VD level of 36.4 ± 7.4 ng/mL. Conversely, within the High Risk category, the prevalence of VD insufficiency rose to 71.8%, reflecting a significantly lower mean VD level (27.0 ± 6.4 ng/mL). A strong linear association and a significant linear trend were confirmed between the risk levels and actual VD status (*χ*^2^ = 27.43, *p* < 0.001; *p*-for-trend < 0.001).

For Overweight Subjects (Figures 1C,D), the scorecard exhibited an even more pronounced discrimination across the risk gradient. In the Low Risk category, the vitamin D sufficiency rate was 90.0% (34.8 ± 5.3 ng/mL). Upon escalating to the High Risk category, the prevalence of VD insufficiency surged to 73.3%, recording the lowest mean serum VD level in the study (24.0 ± 6.3 ng/mL). This risk progression showed a statistically significant association and a highly significant linear trend (*χ*^2^ = 26.64, *p* < 0.001; *p*-for-trend <0.001). These results indicate that both simplified scorecards demonstrated progressive risk stratification into three distinct categories of VD insufficiency.

## 4 Discussion

This cross-sectional study evaluated the prevalence and risk factors of VD insufficiency among Paraguayan young adults and developed a preliminary population-specific, non-invasive risk screening scorecard. Our principal findings reveal a high prevalence of VD insufficiency (48.2%) within this young cohort despite high local solar irradiance. Multivariable modeling demonstrated that female sex, full body clothing coverage, and infrequent mate/tereré consumption were independent risk factors for VD insufficiency, whereas vigorous physical activity was independently associated with lower odds of VD insufficiency, while muscle mass dynamics were associated with higher odds of insufficiency.

The inclusion of sex-specific median centered Δ Muscle Mass as a primary explanatory predictor represents a novel contribution of this study. While conventional screening tools focus primarily on detecting muscle loss in sarcopenia, our findings align with personalized medicine approaches demonstrating that VD dynamics vary across biological subtypes (9, 10). We revealed that non-obese young individuals with higher muscle mass present an elevated relative risk of VD insufficiency. Physiologically, vitamin D receptors (VDR) are highly expressed in skeletal muscle cells, suggesting that a larger volume of active muscle tissue may increase the metabolic sequestration of circulating 25(OH)D, thereby accelerating its tissue clearance (26). Incorporating Δ Muscle Mass prevents this physically active or athletic cohort from being misclassified as low risk by conventional clinical metrics.

An apparently paradoxical pattern emerged: subjects with an athletic phenotype tended to present lower serum 25(OH)D levels, even though vigorous physical activity served as an overall protective factor in multivariable modeling. Biologically, while physical activity often correlates with outdoor solar exposure, the tissue metabolic sequestration exerted by elevated muscle mass (26) might exceed the protective contribution of residual cutaneous synthesis. However, the lack of objective ultraviolet B (UVB) radiation measurement remains an important factor to be evaluated in future studies. Regarding lifestyle factors, the degree of sun exposure via habitual clothing demonstrated a strong direct association with VD insufficiency, displacing simple self-reported sun exposure duration. This indicates that endogenous cutaneous synthesis is substantially impeded when the dermal surface is covered, regardless of ambient solar radiation levels.

A finding of particular ethnographic relevance is the protective role of frequent mate or tereré consumption (≥4 days/week). Epidemiologically, daily mate consumption has been documented to improve serum lipid profiles (27–29). Consistent with this, subgroup analysis revealed a trend toward an inverse correlation between regular mate/tereré consumption and serum triglyceride levels (*p* = 0.061). Recent molecular studies suggest that metabolic dysregulation causally reduces circulating 25(OH)D levels, positioning VD insufficiency as a secondary marker of metabolic stress (30). Given these metabolic benefits, this deeply rooted cultural habit may act as a physiological shield, indirectly curbing the depletion of circulating VD levels. Crucially, however, due to the observational, cross-sectional design of this study, direct causality cannot be inferred, and reverse causality or bidirectional relationships remain possible. Furthermore, sharing mate/tereré in Paraguay is predominantly an outdoor, communal social activity; thus, residual confounding related to unmeasured sun exposure patterns, outdoor leisure time, or broader healthy lifestyle behaviors cannot be excluded. Consequently, frequent mate/tereré consumption may also serve as a surrogate marker for an outdoor, socially active lifestyle favoring cutaneous VD synthesis rather than exerting a purely direct physiological effect.

Through multivariable logistic regression, we established a predictive scorecard tailored to Paraguayan youth. While an alternative model excluding BMI demonstrated a marginally superior statistical fit (AIC = 223.8 vs. 225.7, Supplementary Table S2), BMI was deliberately retained due to its clinical accessibility and practicality in primary healthcare settings (4, 8, 19). Multivariable modeling indicated that while BMI exerted a negligible effect in the overall population (OR = 1.01), higher BMI substantially increased the risk of insufficiency within the overweight subgroup (OR = 1.14). Model performance evaluated via internal validation demonstrated acceptable discrimination in the total population (optimism-corrected AUC = 0.714) and improved discriminative performance when restricted to the overweight subgroup (optimism-corrected AUC = 0.768), supported by a clear risk gradient across risk categories.

Several limitations must be acknowledged when interpreting the findings and clinical applicability of this study. First, the cross-sectional design precludes establishing causal relationships between the identified lifestyle/anthropometric predictors and serum 25(OH)D levels. Second, study recruitment was restricted to a single university institution (USCA) in Caaguazú, Paraguay, during a specific time frame, which introduces potential selection bias and does not account for seasonal variations in ambient solar radiation. Third, body composition parameters were assessed using bioelectrical impedance analysis (BIA) rather than reference imaging techniques such as dual-energy X-ray absorptiometry (DXA), although BIA represents a widely accepted and practical clinical tool. Fourth, while the overall sample size was adequate, the restricted event count in the overweight subgroup (*n* = 68, 29 events) constrained EPV ratio, even after enhancing model parsimony by omitting the non-significant sex variable. To address potential model optimism and overfitting under these EPV constraints, we performed internal validation using 1,000 bootstrap resamples, which demonstrated acceptable discrimination and internal stability. Nevertheless, this subgroup analysis should be interpreted as an exploratory, proof-of-concept pilot assessment. Consequently, this study serves as a baseline screening structure, requiring further validation in larger, multi-center prospective cohorts with broader sample sizes. Fifth, candidate predictor selection partly relied on initial univariable statistical significance screening. Although this criterion was combined with AIC minimization, collinearity diagnostics (VIF < 1.35), and biological plausibility, univariable pre-screening can introduce selection bias, model instability, and overfitting under modern prediction modeling standards (31, 32). Future prospective validation studies should consider pre-specified predictor sets or penalized regression approaches (e.g., LASSO) (33) to minimize data-driven selection bias.

In conclusion, this simplified scorecard—integrating culturally deeply rooted habits such as mate/tereré intake alongside muscle mass dynamics and conventional anthropometrics—offers a practical, low-cost preliminary screening tool for the early identification of VD insufficiency in Paraguayan youth. Given the preliminary nature of this model, external validation in independent populations is strictly required prior to routine clinical implementation.

## Data Availability

The datasets generated and analyzed during the current study are available in the Zenodo repository at https://doi.org/10.5281/zenodo.21960695.

## Footnotes

1 Zenodo: https://doi.org/10.5281/zenodo.21960695.
