# Supplementary material for: Prevalence and non-invasive predictive models of vitamin D insufficiency in Paraguayan youth: the role of body composition and traditional mate consumption
Source: Front Nutr. 2026 Sep 11;13:1953400. doi: 10.3389/fnut.2026.1953400 (PMC13612359; doi:10.3389/fnut.2026.1953400)
Supplement: Supplementary file 1 [file Table_1.DOCX]

Supplementary Material

# 1 Supplementary Data

**Table S1. Association between sociodemographic and anthropometric factors and vitamin D levels.**

| Variables | Category or unit | VD value  (ng/mL) | Welch’s *t*-test | Pearson correlation  coefficient | | VD Insufficiency  (< 30 ng/mL) | VD Sufficiency  (≥ 30 ng/mL) | Chi-square test | | |
| --- | --- | --- | --- | --- | --- | --- | --- | --- | --- | --- |
|  |  | Mean ± SD | *p*-value | Pearson's *r* | *p*-value | *n* (%) | *n* (%) | *χ^2^* | Cramer's *V* | *p*-value |
|  | **Total (*n* = 164)** | **30.7 ± 9.0** |  |  |  | **79 (48.2%)** | **85 (51.8%)** |  |  |  |
| Age (17–35 years) |  |  |  | −0.111 | 0.157 |  |  |  |  |  |
| Sex | Female (*n* = 130) | 29.9 ± 9.1 | < 0.01 | −0.191 | < 0.05 | 70 (53.1%) | 60 (46.9%) | 8.1 | 0.22 | < 0.005 |
|  | Male (*n* = 34) | 34.1 ± 7.8 |  |  |  | 9 (26.5%) | 25 (73.5%) |  |  |  |
| Weight (kg) |  |  |  | 0.003 | 0.966 |  |  |  |  |  |
| Stature (m) |  |  |  | 0.117 | 0.137 |  |  |  |  |  |
| BMI (kg/m^2^) | Total |  |  | −0.070 | 0.356 |  |  |  |  |  |
|  | Overweight (*n* = 68) | 30.8 ± 7.6 |  | −0.268 | < 0.05 | 29 (42.6%) | 39 (57.4%) | 1.4 | 0.093 | 0.233 |
|  | Normal weight (*n* = 96) | 30.7 ± 10.0 |  | 0.035 | 0.735 | 50 (52.1%) | 46 (47.9%) |  |  |  |
| Body fat (%)^*^ | Total |  |  | −0.151 | 0.053 |  |  |  |  |  |
|  | Overweight (*n* = 68) |  |  | −0.336 | < 0.01 |  |  |  |  |  |
|  | Normal weight (*n* = 96) |  |  | −0.091 | 0.381 |  |  |  |  |  |
| Δ Body fat (%)^†^ | Total |  |  | −0.045 | 0.565 |  |  |  |  |  |
|  | Overweight (*n* = 68) |  |  | −0.268 | < 0.05 |  |  |  |  |  |
|  | Normal weight (*n* = 96) |  |  | 0.042 | 0.682 |  |  |  |  |  |
| Muscle mass (%)^*^ | Total |  |  | 0.199 | < 0.05 |  |  |  |  |  |
|  | Overweight (*n* = 68) |  |  | 0.305 | < 0.05 |  |  |  |  |  |
|  | Normal weight (*n* = 96) |  |  | 0.159 | 0.122 |  |  |  |  |  |
| Δ Muscle mass (%)^†^ | Total |  |  | 0.046 | 0.561 |  |  |  |  |  |
|  | Overweight (*n* = 68) |  |  | 0.007 | 0.954 |  |  |  |  |  |
|  | Normal weight (*n* = 96) |  |  | 0.070 | 0.500 |  |  |  |  |  |
| Waist Circumference (cm)^*^ | Total |  |  | −0.048 | 0.539 |  |  |  |  |  |
|  | Overweight (*n* = 68) |  |  | −0.161 | 0.191 |  |  |  |  |  |
|  | Normal weight (*n* = 96) |  |  | 0.003 | 0.974 |  |  |  |  |  |
| Δ Waist Circumference (cm) ^†^ | Total |  |  | −0.182 | < 0.05 |  |  |  |  |  |
|  | Overweight (*n* = 68) |  |  | −0.332 | < 0.01 |  |  |  |  |  |
|  | Normal weight (*n* = 96) |  |  | −0.145 | 0.160 |  |  |  |  |  |
| Neck Circumference (cm)^*^ | Total |  |  | 0.115 | 0.141 |  |  |  |  |  |
|  | Overweight (*n* = 68) |  |  | 0.061 | 0.62 |  |  |  |  |  |
|  | Normal weight (*n* = 96) |  |  | 0.170 | 0.098 |  |  |  |  |  |
| Δ Neck Circumference (cm) ^†^ | Total |  |  | −0.016 | 0.837 |  |  |  |  |  |
|  | Overweight (*n* = 68) |  |  | −0.124 | 0.315 |  |  |  |  |  |
|  | Normal weight (*n* = 96) |  |  | 0.048 | 0.644 |  |  |  |  |  |
| Cholesterol (mg/dL) |  |  |  | −0.097 | 0.217 |  |  |  |  |  |
| Triglyceride (mg/dL) |  |  |  | −0.076 | 0.331 |  |  |  |  |  |
| HDL (mg/dL) |  |  |  | 0.054 | 0.491 |  |  |  |  |  |

*: There is a significant correlation between the sex of the participants and the following anthropometric variables: Body Fat (*r* = 0.654, *p* < 0.001), Muscle Mass (*r* = –0.832, *p* < 0.001), Waist Circumference (*r* = −0.423, *p* < 0.001), and Neck Circumference (*r* = −0.620, *p* < 0.001). ^†^: To eliminate potential confounding and baseline gender bias inherent to crude anthropometric measurements, the absolute values of body fat, muscle mass, waist circumference, and neck circumference were transformed into sex-specific median deviations, designated as delta (Δ) variables. For each participant, the Δ variable was calculated by subtracting their respective sex-specific median from their absolute measured value: Δ Variable = Absolute Individual Value – Sex-Specific Median. The reference median values used for this transformation were derived from the study population (*n* = 164) and established as follows: Body Fat: Females = 37.7%, Males = 23.3%, Muscle Mass: Females = 25.5%, Males = 38.7%, Waist Circumference: Females = 75.0 cm, Males = 93.5 cm, Neck Circumference: Females = 33.0 cm, Males = 38.0 cm. Following the transformation to difference variables, Δ Body Fat (%), Δ Muscle Mass (%), and Δ Neck Circumference showed no significant correlations with sex (*r* = 0.014, *p* = 0.858, *r* = 0.034, *p* = 0.663, and *r* = 0.064, *p* = 0.417, respectively), whereas Δ Waist Circumference maintained a quantitatively lower but statistically significant correlation (*r* = 0.245, *p* < 0.005). Additionally, BMI showed significant correlations with Body Fat (*r* = 0.693, *p* < 0.001), Δ Body Fat (*r* = 0.864, *p* < 0.001), Muscle Mass (*r* = −0.344, *p* < 0.001), and Δ Muscle Mass (*r* = −0.326, *p* < 0.005), Waist Circumference (*r* = 0.655, *p* < 0.001), and Δ Waist Circumference (*r* = 0.728, *p* < 0.001)

Table S2. Logistic regression between vitamin D insufficiency and body composition

| Variable | Model A | | | Model B | | | Model C | | | Model D | | | Model E | | | Model F | | |
| --- | --- | --- | --- | --- | --- | --- | --- | --- | --- | --- | --- | --- | --- | --- | --- | --- | --- | --- |
|  | OR  (95% CI) | *p*-value | VIF | OR  (95% CI) | *p*-value | VIF | OR  (95% CI) | *p*-value | VIF | OR  (95% CI) | *p*-value | VIF | OR  (95% CI) | *p*-value | VIF | OR  (95% CI) | *p*-value | VIF |
| Sex  (Male vs. Female)^*^ | 3.13  (1.29–7.55) | 0.011 | 1.08 | 3.02  (1.26–7.24) | 0.013 | 1.07 | 3.62  (1.54–8.52) | 0.003 | 1.05 | 3.51  (1.50–8.21) | 0.004 | 1.03 | 3.50  (1.49–8.20) | 0.004 | 1.04 | 3.57  (1.52–8.37) | 0.003 | 1.04 |
| BMI (kg/m^2^)^*^ | 0.92  (0.77–1.09) | 0.324 | 5.60 | 0.95  (0.84–1.08) | 0.420 | 2.93 | 0.97  (0.84–1.12) | 0.679 | 4.06 | 1.01  (0.94–1.10) | 0.737 | 1.11 | - | - | - | - | - | - |
| Δ Body Fat (%) | 1.03  (0.94–1.13) | 0.558 | 4.52 | - | - | - | 1.03  (0.94–1.13) | 0.490 | 4.47 | - | - | - | - | - | - | 1.02  (0.97–1.07) | 0.516 | 1.22 |
| Δ Waist Circumference (cm) | 1.03  (0.98–1.09) | 0.219 | 2.87 | 1.04  (0.98–1.09) | 0.201 | 2.84 | - | - | - | - | - | - | - | - | - | - | - | - |
| Δ Muscle Mass (%)^*^ | 1.03  (0.98–1.10) | 0.264 | 1.28 | 1.03  (0.97–1.09) | 0.326 | 1.14 | 1.04  (0.98–1.10) | 0.228 | 1.28 | 1.03  (0.97–1.09) | 0.298 | 1.15 | 1.03  (0.97–1.08) | 0.324 | 1.04 | 1.04  (0.98–1.10) | 0.516 | 1.04 |
| Omnibus test  (*p*-value) | 0.040 | | | 0.024 | | | 0.041 | | | 0.024 | | | 0.009 | | | 0.020 | | |
| AIC | 227.5 | | | 225.9 | | | 227.2 | | | 225.7 | | | 223.8 | | | 225.3 | | |

OR = Odds Ratio; 95% CI = 95% Confidence Interval; VIF = Variance Inflation Factor; AIC = Akaike Information Criterion. Model D was selected as the baseline structure for the predictive score due to the elimination of redundancy with BMI and the optimization of the overall fit (*p* = 0.024, AIC = 225.7). ^*^: Based on these results, the three factors (Sex, BMI, and Δ Muscle Mass) were selected for further logistic regression.
